# Outcomes of Simultaneous Resections and Classical Strategy for Synchronous Colorectal Liver Metastases in Sweden: A Nationwide Study with Special Reference to Major Liver Resections

**DOI:** 10.1007/s00268-020-05475-5

**Published:** 2020-03-17

**Authors:** Valentinus T. Valdimarsson, Ingvar Syk, Gert Lindell, Per Sandström, Bengt Isaksson, Magnus Rizell, Agneta Norén, Bjarne Ardnor, Christian Sturesson

**Affiliations:** 1 Department of Clinical Sciences Lund, Surgery, Skane University Hospital, Lund University, Lund, Sweden; 2 Department of Clinical Sciences Malmö, Surgery, Skane University Hospital, Lund University, Malmö, Sweden; 3 Department of Surgery, Linköping University, Linköping, Sweden; 4 Department of Clinical and Experimental Medicine, Linköping University, Linköping, Sweden; 5 Department of Surgical Sciences, Uppsala University, Uppsala, Sweden; 6 Department of Transplantation and Liver Surgery, Sahlgrenska Academy, University of Gothenburg, Gothenburg, Sweden; 7 Department of Surgery, Umeå University Hospital, Umeå, Sweden; 8 Division of Surgery, Department of Clinical Science, Intervention and Technology (CLINTEC), Karolinska Institutet, Karolinska University Hospital, 141 86 Stockholm, Sweden

## Abstract

**Background:**

About 20% of patients with colorectal cancer have liver metastases at the time of diagnosis, and surgical resection offers a chance for cure. The aim of the present study was to compare outcomes for patients that underwent simultaneous resection to those that underwent a staged procedure with the bowel-first (classical) strategy by using information from two national registries in Sweden.

**Methods:**

In this prospectively registered cohort study, we analyzed clinical, pathological, and survival outcomes for patients operated in the period 2008–2015 and compared the two strategies.

**Results:**

In total, 537 patients constituted the study cohort, where 160 were treated with the simultaneous strategy and 377 with the classical strategy. Patients managed with the simultaneous strategy had less often rectal primary tumors (22% vs. 31%, *p* = 0.046) and underwent to a lesser extent a major liver resection (16% vs. 41%, *p* < 0.001), but had a shorter total length of stay (11 vs. 15 days, *p* < 0.001) and more complications (52% vs. 36%, *p* < 0.001). No significant 5-year overall survival (*p* = 0.110) difference was detected. Twenty-five patients had a major liver resection in the simultaneous strategy group and 155 in the classical strategy group without difference in 5-year overall survival (*p* = 0.198).

**Conclusion:**

Simultaneous resection of the colorectal primary cancer and liver metastases can possibly have more complications, with no difference in overall survival compared to the classical strategy.

## Introduction

Cancer from the colon or rectum is the third most common malignancy worldwide [1, 2]. At diagnosis, 15–20% of patients present with synchronous liver metastases (sCRLM) [3–5]. Although possible for only a minority of patients, resection of all tumors offers a chance for cure. Different strategies exist for surgical treatment. The primary tumor can be resected first, and the liver metastases can be addressed at a later stage, with or without chemotherapy in the interval between operations, the so-called classical strategy. Alternatively, the surgical treatment order is reversed, where the liver metastases are resected before the primary, the liver-first strategy [6]. The third option includes resection of both the primary colorectal cancer and the metastases in the liver during the same operation, the simultaneous strategy.

The simultaneous strategy seems to be safe when compared to the other strategies and has been shown to reduce the total length of hospital stay. This strategy is increasingly applied when a patient has a limited liver tumor disease burden, and the primary tumor resection is assumed uncomplicated. The indication for proposing the simultaneous strategy is still evolving. No overall survival difference has been observed between the above strategies, although most studies only analyze patients that complete the entire surgical strategy without intention-to-treat analysis [7–10]. No randomized controlled investigation exists to date.

The treatment impact on patient outcome has previously been analyzed only from retrospective data collected from single or a few centers. No nationwide study has previously been published on simultaneous resections, and little data are available for patients that have undergone major resections. The aim of this study was to evaluate and compare a simultaneous strategy to a classical strategy for patients diagnosed with sCRLM, based on information from two population registries from Sweden with a focus on patients undergoing major liver resections.

## Material and methods

National registries for colorectal (SCRCR) and liver and bile duct (Sweliv) cancers were used to identify patients diagnosed with colorectal cancer between 2008 and 2015. Both registries have prospective registration of data, where SCRCR includes 94–98% of all patients diagnosed with colorectal cancer in Sweden [11], and Sweliv includes patients with primary liver cancer and bile duct cancer in addition to all surgical treatments for a primary and metastatic cancer in the liver. Sweliv includes 96% of all patients with cancers in the liver or bile ducts in Sweden [12].

Patients with colorectal cancer and liver metastases were identified from the registries at the time of diagnosis and are defined as having sCRLM. Patients that underwent an acute operation for their primary were excluded. A cohort of patients only undergoing elective colorectal resection and no liver resection was analyzed separately. Patients undergoing colorectal resection within 6 months from the diagnosis, and both primary tumor and liver resection within 12 months from diagnosis, constituted the patient cohort in the present study. Patients with sCRLM that only underwent liver resection and those that underwent the liver-first strategy were identified separately, as previously published [13]. A comparison was made between patients that had undergone the simultaneous strategy and those that underwent the classical strategy. A liver resection of three or more Couinaud’s segments was classified as a major resection. Morbidity was registered as 30-day complications demanding treatment. A more detailed specification of morbidity was not available in the registries. In the staged resection group, a complication in either procedure was considered a complication for the procedures combined. An R0 surgical margin is defined as a microscopic surgical-free specimen margin. Tumor burden score (TBS) in the liver was calculated as TBS^2^ = *d*^2^ + *n*^2^, where *d* = largest liver tumor diameter (cm) and *n* = number of liver lesions [13].

### Statistics

Results were showed as numbers and percentages when categorical variables and Fisher’s exact test were used to compare groups. For continuous variables, results are presented as median with interquartile ranges (IQRs), and Mann–Whitney *U* test was used to compare groups. Survival from the time of diagnosis was estimated using Kaplan–Meier analysis. Patient and tumor characteristics effect on survival was investigated using multi- and univariate Cox proportional hazards (PH) models. A *p* value of less than 0.05 was considered statistically significant. The statistical software used was R (R Core Team (2018). R Foundation for Statistical Computing, Vienna, Austria).

## Results

**Fig. 1:**
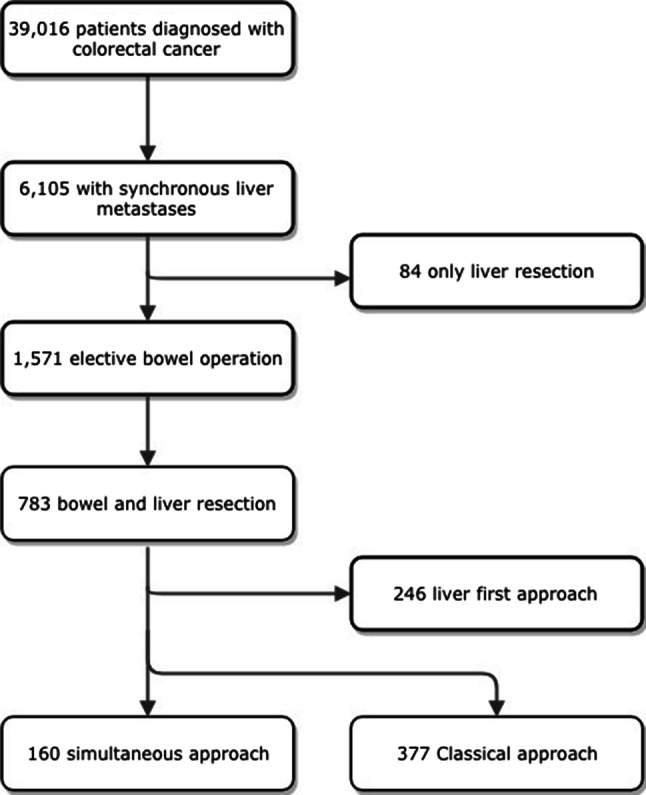
Flowchart of the patient selection process

In the SCRCR, 39 016 patients with colorectal cancer were identified, of which 6105 (16%) patients had liver metastases at the time of diagnosis. Of those, a total of 1571 (26%) underwent elective surgery of the primary tumor. Seven-hundred and eighty-eight patients underwent colorectal resection only and no liver resection. A total of 783 patients underwent both colorectal and liver resections, constituting 2% of the initially identified patient group diagnosed with colorectal cancer. Of the 783 patients, 377 underwent a classical strategy, and 160 underwent a simultaneous strategy, as shown in Fig. 1. Patients’ characteristics for the classical and simultaneous strategy are shown in Table 1. One patient died within 30 days of the resection in the simultaneous groups, but none in the classical strategy group. The number resected with the simultaneous strategy increased in the first 3 years of the study period from six patients per year to 30 per year, with a median of 26 (18–30) patients resected per year. Eighteen patients (5%) underwent a laparoscopic liver procedure in the classical strategy group and 4 (3%) in the simultaneous strategy group (*p* = 0.340).

**Table 1:** Clinical features of patients

	Simultaneous strategy	Classical strategy	*p*^‡^
Patients	160	377	
Male gender	90 (56)	234 (62)	0.211
Age (years)*	65 (58–72)	66 (58–73)	0.396
American Society of Anesthesiologists (3–4)	32 (20)	74 (20)	0.906^§^
Body mass index (kg/m^2^) *	25 (22–28)	25 (23–28)	0.434^§^
Preoperative radiotherapy	29 (18)	84 (22)	0.300
Neoadjuvant chemotherapy	101 (64)	274 (73)	0.029
Localization (rectum)	35 (22)	115 (31)	0.046
T4 primary	41 (26)	85 (23)	0.435
Lymphatic node-positive, primary tumors	105 (66)	264 (70)	0.411
Number of liver tumors*	2 (1–4)	2 (1–4)	1^§^
Liver tumor size (mm)*	20 (12–30)	20 (14–35)	0.202^§^
Tumor burden score*	3.2 (2.1–4.5)	3.6 (2.2–4.2)	0.500^§^
Portal vein embolization	0 (0)	15 (4)	0.008
Major liver surgery	25 (16)	152 (41)	<0.001
Radical liver resection	145 (91)	350 (93)	0.370
Total loss of blood (ml)*	600 (250–950)	850 (474–1456)	<0.001^§^
Complications, demanding treatment	84 (52)	136 (36)	<0.001
Total length of stay (days)*	11 (8–15)	15 (12–20)	<0.001^§^

Percentages are in parentheses unless otherwise indicated: *median (interquartile range). ^‡^*Chi-square* test, except ^§^Mann–Whitney *U* test

Twenty-five and 155 patients underwent a major liver resection in the simultaneous and classical strategy group, respectively (Table 2). A total of 135 patients underwent a minor liver resection in the simultaneous group and 222 in the classical group. The simultaneous group undergoing minor liver resections had: less often a rectal primary (5 vs. 33%, *p* < 0.001), less intraoperative blood loss (600 (300–900) vs. 700 (350–1250) ml, *p* = 0.003), and shorter total length of stay (11 (7–15) vs. 16 (14–20) days, *p* < 0.001).

**Table 2:** Clinical features of patients that underwent a major liver resection

	Simultaneous strategy	Classical strategy	*p*^‡^
Patients	25	155	
Male gender	12 (48)	98 (63)	0.185
Age (years)*	63 (59–68)	65 (58–69)	0.820^§^
American Society of Anesthesiologists (3–4)	5 (20)	31 (17)	0.576
Body mass index (kg/m^2^) *	25 (24–26)	25 (23–27)	0.828^§^
Preoperative radiotherapy	3 (12)	29 (19)	0.576
Neoadjuvant chemotherapy	21 (84)	127 (82)	0.770
Localization (rectum)	6 (24)	41 (26)	0.878
T4 primary	3 (12)	39 (25)	0.419
Lymphatic node-positive, primary	20 (80)	115 (75)	0.802
Number of liver tumors*	3 (2–4)	3 (2–4)	0.985
Liver tumor size (mm)*	31 (20–59)	25 (15–43)	0.119^§^
Tumor burden score*	5.7 (3.2–7.2)	4.5 (3.3–6.4)	0.408^§^
Portal vein embolization	0 (0)	15 (10)	0.222
Radical liver resection	24 (96)	143 (93)	1
Total loss of blood (ml)*	1100 (500–1600)	1100 (630–1800)	0.6393^§^
Complications, demanding treatment	11 (44)	57 (37)	0.5109
Total length of stay (days)*	11 (9–15)	17 (14–21)	<0.001^§^

Percentages are in parentheses unless otherwise indicated: *median (interquartile range). ^‡^*Chi-square* test, except ^§^Mann–Whitney *U* test

For patients experiencing complications, the simultaneous strategy resulted in a shorter total hospital length of stay (13 (10–17) vs. 20 (15–28) days, *p* < 0.001), as compared to the classical strategy. A logistic multivariate odds ratio for complications was significantly increased for simultaneous treatment but decreased for female gender (Table 3).

**Table 3:** Uni- and multivariate logistic regression for complications needing treatment

	Univariate analysis		Multivariate analysis	
Treatment				
Classical strategy	Reference		Reference	
Simultaneous strategy	2.52 (1.72–3.69)	<0.001*	2.23 (1.39–3.58)	<0.001*
Age (years)				
<65	Reference		Reference	
≥65	0.91 (0.64–1.29)	0.579	0.86 (0.57–1.29)	0.461
Gender				
Male	Reference		Reference	
Female	0.67 (0.46–0.96)	0.03*	0.65 (0.43–0.98)	0.041*
American Society of Anesthesiologists				
1–2	Reference		Reference	
3–4	1.43 (0.93–2.21)	0.103	1.36 (0.84–2.2)	0.212
Body mass index (kg/m^2^)				
<25	Reference		Reference	
≥25	0.94 (0.66–1.36)	0.751	0.98 (0.65–1.46)	0.904
Tumor localization				
Colon primary	Reference		Reference	
Rectal primary	1.44 (0.98–2.12)	0.061	0.81 (0.37–1.67)	0.573
Liver resection size				
Minor	Reference		Reference	
Major	0.73 (0.5–1.06)	0.103	0.8 (0.51–1.24)	0.318
Radiotherapy				
No	Reference		Reference	
Yes	1.93 (1.27–2.94)	0.002*	1.89 (0.84–4.45)	0.134
Bleeding				
<600 ml	Reference		Reference	
≥600 ml	1.09 (0.72–1.63)	0.692	1.04 (0.66–1.62)	0.863
Neoadjuvant chemotherapy				
No	Reference		Reference	
Yes	2.11 (1.47–3.04)	<0.001*	1.52 (0.95–2.41)	0.080

Data are presented as odds ratios with a 95% confidence interval in parenthesis. *p< 0.05

**Fig. 2:**
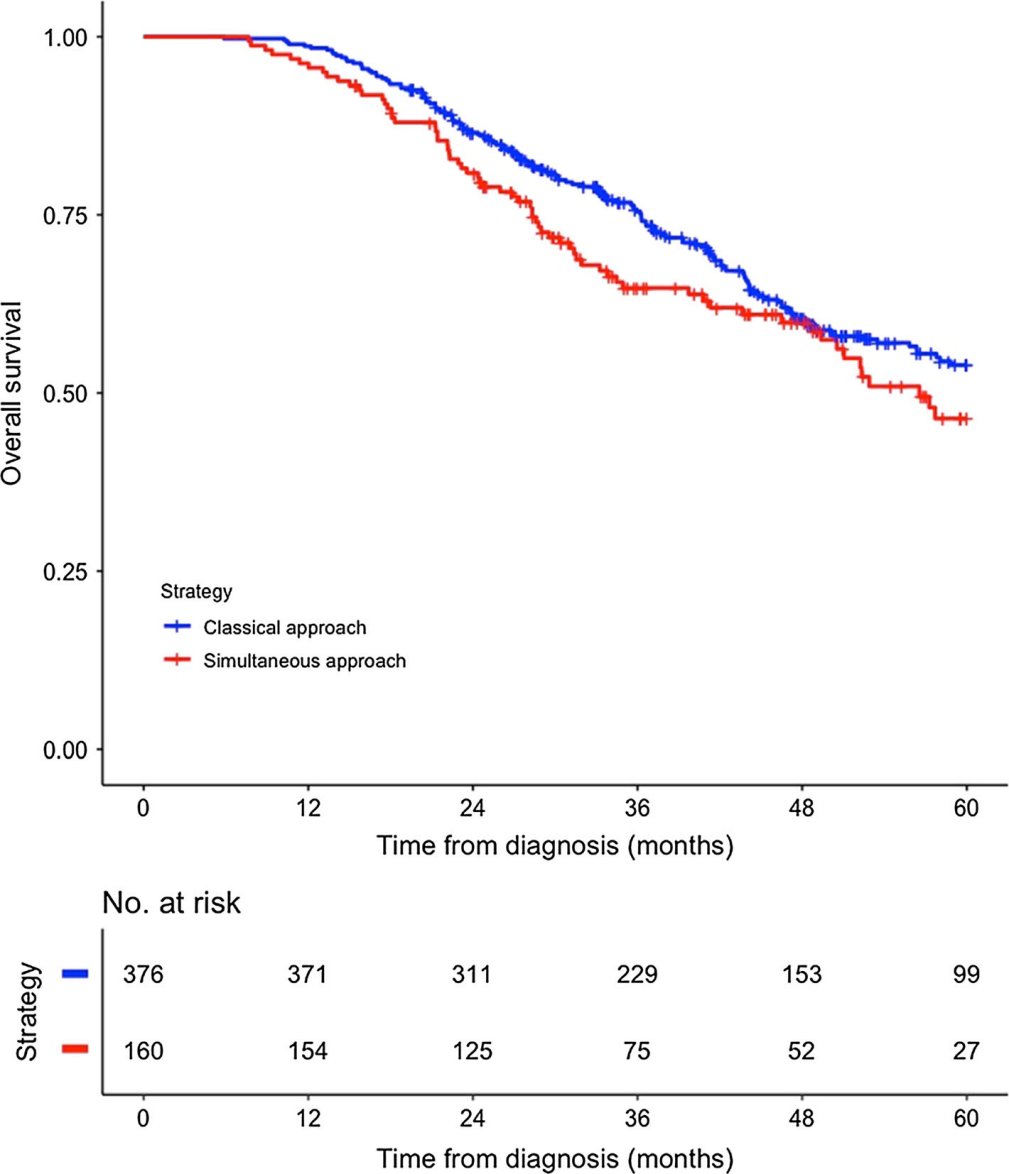
Overall survival from diagnosis for resected patients with synchronous liver metastases, *p* = 0.110 (log-rank test)

The median follow-up time was 41 (27–58) months, and overall survival did not differ between groups (*p* = 0.110), with a 5-year survival from diagnosis of 54% in the classical strategy group and 46% in the simultaneous strategy group, and median survival was 49 and 58 months, respectively, as shown in Fig. 2. At the end of the study, a total of 231 patients were deceased. For the classical strategy group, the interval between the procedures was 4.7 (2.8–6.1) months. Analyzing the patients undergoing a major liver resection, no difference in overall survival was found between the simultaneous and staged groups (*p* = 0.198).

In Table 4, the results from uni- and multivariate Cox PH models are shown. The multivariate analysis showed higher ASA class, higher liver tumor burden, and T4 primary tumor to be risk factors for decreased overall survival but not simultaneous or staged operation.

**Table 4:** Cox proportional hazards analysis for overall survival

	Univariate HR		Multivariate HR	
Treatment				
Classical strategy	Reference		Reference	
Simultaneous strategy	0.79 (0.59–1.05)	0.107	0.83 (0.6–1.14)	0.243
Age (in years)				
<60	Reference		Reference	
≥60–70	0.78 (0.56–1.09)	0.140	0.78 (0.54–1.13)	0.189
≥70	1.15 (0.83–1.6)	0.407	1.18 (0.81–1.7)	0.392
Gender				
Male	Reference		Reference	
Female	0.83 (0.62–1.1)	0.199	0.9 (0.66–1.22)	0.495
Lymphatic node primary tumor				
Negative	Reference		Reference	
Positive	1.55 (1.13–2.12)	0.007*	1.26 (0.9–1.75)	0.182
pT4 primary tumor				
No	Reference		Reference	
Yes	1.92 (1.43–2.57)	<0.001*	1.84 (1.34–2.54)	<0.001*
Primary tumor localization				
Colon	Reference			
Rectum	0.98 (0.73–1.31)	0.877		
Liver resection size				
Minor	Reference			
Major	1.1 (0.83–1.46)	0.494		
Tumor burden score				
<3	Reference		Reference	
≥3–9	1.67 (1.21–2.29)	0.002*	1.73 (1.24–2.41)	0.001*
≥9	2.99 (1.86–4.81)	<0.001*	3.04 (1.83–5.04)	<0.001*
Liver tumor numbers				
<3	Reference			
≥3	1.53 (1.16–2.02)	0.003*		
Liver tumor size (cm)				
<5	Reference			
≥5	1.92 (1.33–2.77)	<0.001*		
American Society of Anesthesiologists				
1–2	Reference		Reference	
3–4	1.47 (1.07–2.02)	0.019*	1.52 (1.09–2.13)	0.014*
Body mass index (kg/m^2^)				
<25	Reference			
≥25–35	1.09 (0.82–1.46)	0.545		
≥35	1.39 (0.61–3.18)	0.436		

Data are presented as hazard ratios (HR) with a 95% confidence interval (CI) in parenthesis. *p < 0.05

The group undergoing colorectal resection only (that is, without subsequent liver resection) was older (72 (64–79) years, *p* < 0.001) and had more frequently: T4 primary tumors (291 (37%), *p* = 0.010), lymphatic node-positive primaries (630 (82%), *p* < 0.001), and ASA score of 3–4 (228 (29%), *p* = 0.027), compared to the simultaneous group. The primary only group received less often neoadjuvant chemotherapy (59 (7%), *p* < 0.001) and radiation therapy (83 (11%), *p* = 0.01), compared to the simultaneous group. The primary only group had 11% 5-year overall survival and a median survival of 15 months.

## Discussion

The aim of the present study was to compare the simultaneous strategy to the classical strategy for all patients diagnosed with sCRLM, using data from two national quality cancer registries from Sweden. It was found that 16% of the patients diagnosed with colorectal cancer had sCRLM, which is in line with previous studies [14]. About 50% of the patients that underwent elective colorectal resections did not undergo liver resections. The reason these patients did not undergo liver resections is unknown, but symptomatic palliative resections probably account for some of the resections. Palliative colorectal resection rates in patients with sCRLM have been reported to be between 16 and 60% [15–17]. As the data used for the present study do not allow identification of palliative resections, no analysis by intention to treat could be made. The reason for not undergoing liver resections could also be that a patient with a curative intention did not finish the planned strategy, i.e., liver and colorectal. It has been shown that about 35% of patients diagnosed with sCRLM do not finish a planned liver and colorectal resection, regardless of the treatment strategy, mostly due to disease progression [18].

No differences regarding gender, age, ASA score, BMI, T4 primary stage, lymph node-positive primaries, or liver tumor burden score were found between the simultaneous and classical strategy groups. However, rectal cancer and major liver resections were more frequent in the classical strategy group. This indicates that patients are selected for either strategy based on the size of the planned liver surgery and whether they have a rectal or colon primary tumor. In a meta-analysis by Gavriilidis et al., no significant pooled difference was found for gender, age, rectal primary, size, or number of liver tumors or complications, but the simultaneous strategy group had a shorter total length of stay, received less often neoadjuvant therapy, and underwent less often major liver resections [19]

Concerning morbidity, it was not possible to separate major morbidity (Clavien–Dindo grade ≥ 3) from minor morbidity in the present study. Despite the higher complication rate in the simultaneous group, the total length of stay was shorter, perhaps pointing to less clinically significant complications. Increased morbidity after simultaneous resections has also been published [20]. In the present study, no difference in survival could be found between groups, as reported in previous studies [19–21].

If a simultaneous procedure significantly shortens the hospital stay from 15 (12–20) to 11 (8–15) days and a cost of a hospital bed in Sweden is USD 914 [22], the cost per patient could be reduced from USD 13,710 (10,968–18,280) to USD 10 054 (7312–13,710).

The use of laparoscopy was rare during the study period, with only 18 patients (5%) in the classical strategy group and 4 patients (3%) in the simultaneous strategy group who underwent laparoscopic liver procedures. Data on the use of laparoscopy for resecting colon and rectal cancer in the classical strategy were missing in the present study. Registry data on colon resections for cancer during 2007–2011 in Sweden showed that 6% of resections were performed laparoscopically [11]. Comparing the groups of patients that underwent major liver resections (simultaneous vs. classical strategy), a significant difference was found for a shorter total length of hospital stay only. No significant 5-year survival difference was found, in line with previously published studies [21, 23–26]. The sample size was, however, small in the simultaneous major liver resection group (*N* = 25), making it difficult to make a definite conclusion on the result.

Cox PH analysis showed that a more advanced colorectal cancer and liver tumor disease, and higher comorbidity negatively affected survival in univariate analysis. On multivariate analysis, an increased tumor burden score, T4 primary stage, and higher comorbidity were related to worse survival, shown in Table 3. These factors have previously been linked to survival [13].

No randomized controlled trial evidence is available to support the use of any strategy for patients with sCRLM. All published studies, therefore, have an intrinsic selection and referral biases [27], as is the case with this study. No multivariate analysis models, such as propensity score matching, retrospective matching, or other regression models, can ever replace randomization. A propensity score matching has gained increased popularity in recent years, with an often unclear matching process. A statistical regression model is often more transparent and can even be more useful and comprehensive when detecting differences in treatment effect [28, 29].

One of the weaknesses of the present study is that the used registries do not allow calculation of recurrence-free survival, nor was it possible to analyze the data according to intention to treat. Another shortcoming of the study is that no data on adjuvant chemotherapy were included. The usage rates of adjuvant chemotherapy have previously not shown to differ between strategies [22]. The strength of this study is that it is based on a national population with a prospective registration.

In conclusion, the present study has shown that patients chosen for simultaneous liver and primary resection had a shorter total length of hospital stay, similar overall survival but can possibly have more complications compared to patients allocated to a classical strategy.
